# Anticoagulation treatment for patients with coronavirus disease 2019 (COVID-19) and its clinical effectiveness in 2020: A meta-analysis study

**DOI:** 10.1097/MD.0000000000027861

**Published:** 2021-11-24

**Authors:** Jingyi Ge, Yingmin Ma, Zhipeng Wu, Jiawei Jin, Xiao Sun

**Affiliations:** a Department of Respiratory Medicine, Beijing Rehabilitation Hospital, Capital Medical University, Beijing, People's Republic of China; b Department of Respiratory and Infections Medicine, Beijing You An Hospital, Capital Medical University, Beijing, People's Republic of China; c Department of Respiratory and Critical Care Medicine, Beijing Chaoyang Hospital, Capital Medical University, The Clinical Research Center, Beijing Chaoyang Hospital, Capital Medical University, Beijing, People's Republic of China; d Department of Epidemiology, Tulane University School of Public Health and Tropical Medicine, New Orleans, LA.

**Keywords:** anticoagulation, bleeding, COVID-19, thrombosis

## Abstract

**Background:**

To better inform efforts to treat and control the current outbreak with effective anticoagulant treatment strategies for coronavirus disease 2019 patients.

**Methods:**

We searched Cochrane Library, Pubmed, EMBASE, MEDLINE, SCIEXPANDED, Web of Science, Google Scholar, CNKI (Chinese Database), WanFang (Chinese Database), CBM (Chinese Database), VIP (Chinese Database) for studies published from November 1, 2019 to October 1, 2020, and we searched references of identified articles. Studies were reviewed for methodological quality. A random-effects model was used to pool results. Heterogeneity was assessed using *I*^2^. Publication bias was assessed using funnel plot.

**Results:**

Fourteen studies involving 7681 patients were included. We meta-analyzed the bleeding, deep vein thrombosis, and pulmonary embolism risk between no anticoagulation and prophylactic anticoagulation, and found no significant difference. The same trend occurred in the comparison between with and without anticoagulation. However, when compared with no anticoagulation, both prophylactic anticoagulation (odd ratio [OR] = 0.80, 95% confidence interval [CI]: 0.69–0.93) and therapeutic anticoagulation (OR = 0.91, 95% CI: 0.80–1.05) had lower risk of mortality. Furthermore, the risk of overall bleeding among patients with therapeutic anticoagulation was 3.11 times (95% CI: 2.29–4.24) than that of patients with prophylactic anticoagulation. On the contrary, therapeutic anticoagulation had lower risk of deep vein thrombosis than prophylactic anticoagulation (OR = 0.34, 95% CI: 0.19–0.63).

**Conclusions:**

Among coronavirus disease 2019 patients, preventive and therapeutic anticoagulation were more beneficial than no anticoagulation for reducing mortality rate. The result will inform healthcare providers and public health policy makers in efforts to treat and control the current outbreak.

## 1 Introduction

Severe acute respiratory syndrome coronavirus 2 (SARS-CoV-2) causes an ongoing global pandemic of coronavirus disease 2019 (COVID-19), leading to more than 135 million infections and mortality rate of 2.1% across the world.^[1]^ Several recent studies have shown that COVID-19 patients are prone to have coagulation disorder which is a major cause of death.^[2–6]^ These abnormal coagulation parameters are results from vascular endothelial cell injury and have been associated with serious thrombotic complications such as deep venous thromboembolism (VTE), pulmonary embolism, myocardial infarction, and cerebral infarction, which will further lead to death, especially among severe COVID-19 cases.^[2,3,7,8]^ Previous report demonstrated that up to 30% COVID-19 patients in intensive care settings suffer from VTE.^[9]^ Several scientific societies and authors, including the American Society of Hematology, International Society on Thrombosis and Haemostasis,^[10]^ CHEST Guideline and Expert Panel,^[11]^ and others have already proposed specific guidelines and recommendations on the use of thromboprophylaxis in patients with COVID-19. Besides, the consensus among Chinese experts on anticoagulation therapy refers to unfractionated heparin/low-molecular-weight heparin, topical citrate anticoagulation, argatroban or bivalirudin, and so on.^[12]^

VTE, arterial thrombosis, and microvascular thrombosis have all been well-described,^[13–16]^ and previous observational cohort studies have provided evidence that use of anticoagulation in patients with COVID-19 was associated with decreased risk of mortality.^[17,18]^ However, these studies always had limitations of small sample size with an imperfect healthcare systems. High VTE rates have been reported in severe COVID-19 patients despite the use of prophylactic anticoagulation.^[13,19]^ Higher level of evidence-based medicine is urgently needed to determine the role of anticoagulant therapy in treating COVID-19 patients.

Although our understanding of the hematologic manifestations of COVID-19 remains in its early stage, this systematic review aimed to provide a summary of current estimates of VTE risk, as well as its association with poor outcomes. Besides, the study discussed benefits and harms of anticoagulation, and provide suggestions for its prevention and management in COVID-19 patients.

## 2 Methods

### 2.1 PICO question

*Population* patients with confirmed COVID-19.

*Intervention* anticoagulant therapy, therapeutic or preventive anticoagulant therapy.

*Comparison* no anticoagulant therapy.

*Outcomes* clinically apparent bleeding, thromboembolism and in-hospital all-cause mortality of COVID-19 patients.

### 2.2 Search strategy and study selection

Our systematic review and meta-analysis were undertaken according to Preferred Reporting Items for Systematic Reviews and Meta-Analyses (PRISMA) guidelines (Table S1, Supplemental Digital Content) and the protocol had been registering in the PROSPERO database (Registration number: CDR42021233116).

A literature search was performed using the electronic database of Cochrane Central Database, PubMed, EMBASE, MEDLINE, SCIEXPANDED, Web of Science, Google Scholar, CNKI (Chinese Database), WanFang (Chinese Database), CBM (Chinese Database), VIP (Chinese Database) from November 1, 2019 to October 1, 2020, with the following search terms: (“coronavirus” or “nCoV” or “SARS-CoV-2” or “COVID-19”) and (“anticoagulant” odd ratio [OR] “anticoagulation” OR “heparin”). We complemented the search by checking the grey literature and cross referencing relevant reviews were identified in current study.

### 2.3 Inclusion and exclusion criteria

Articles were included based on the following characteristics: observational studies or randomized controlled trials of confirmed COVID-19 subjects; medical records were available on anticoagulation treatment and associated outcomes regarding bleeding, thrombosis, or mortality data. The articles were excluded if the articles: focused on patients with pre-condition that may lead to bleeding, previous use of anticoagulant therapy; had overlapped data; had diminutive data volume, such as a sample size less than 10 patients; were reviews, conference reports, case reports, letters to the editor, editorials, and expert opinions.

### 2.4 Data abstraction and quality assessment

Data were collected by 3 independent reviewers (Jingyi Ge, Xiao Sun, and Yingmin Ma) and duplicate articles were deleted by EndNote X8 (Clarivate Analytics (formerly Thomson Reuters), Philadelphia, PA, USA). Then reviewers independently screened the titles and abstracts of the retrieved articles to exclude obvious irrelevant studies. The full text of potentially relevant studies would then be reviewed in accordance with the pre-specified criteria. If authors of the studies were similar or data were extracted from the same database, the study period would be noted. Only the latest study would be included if the study period was overlapped. Any disagreement between authors will be resolved by consensus with a third author (Yingmin Ma). In the case of data missing, we would contact original trial authors requesting for clarifications and more data.

The risk of bias of qualified studies was independently assessed by all reviewers, any discrepancy would be resolved by discussion. Quality assessment of included articles was conducted by Newcastle-Ottawa Scale^[20]^ which consisted of 3 parameters: selection, comparability, and exposure assessment (Table S2, Supplemental Digital Content). The potential risk of bias in each clinical trial would be evaluated by Cochrane collaboration tool^[21]^ through 7 domains (Table S3, Supplemental Digital Content). Each domain would be scored as “low risk”, “high risk”, or “unclear”.

### 2.5 Data analyses

Data synthesis and analysis was performed using STATA (version 15.1; Stata Corporation, College Station, TX). Random-effects meta-analysis was used to calculate an overall proportion or a summary estimate of means of each outcome. They are pooled estimated ratios of component proportion of clinically apparent overall bleeding, thromboembolism (including deep venous thrombosis and pulmonary embolism) and in-hospital all-cause mortality with 95% confidence intervals of different groups (undergoing anticoagulant therapy vs no anticoagulant therapy, therapeutic anticoagulant therapy vs preventive anticoagulant therapy) of COVID-19 patients. To minimize the impact of studies with extremely small or extremely large effectiveness estimates on overall estimates, Freeman-Tukey double arcsine transformation was used to stabilize the variance of specific prevalence rates before conducting random-effects meta-analysis models.

Heterogeneity between studies was assessed using *I*^2^, with threshold values of 25%, 50%, and 75% representing low, moderate, and high heterogeneity, respectively. If substantial heterogeneity (*I*^2^ > 75%) was detected, we further explored the possible source of heterogeneity through subgroup analysis. Publication bias was assessed by funnel plot.^[22]^

## 3 Result

**Figure 1:**
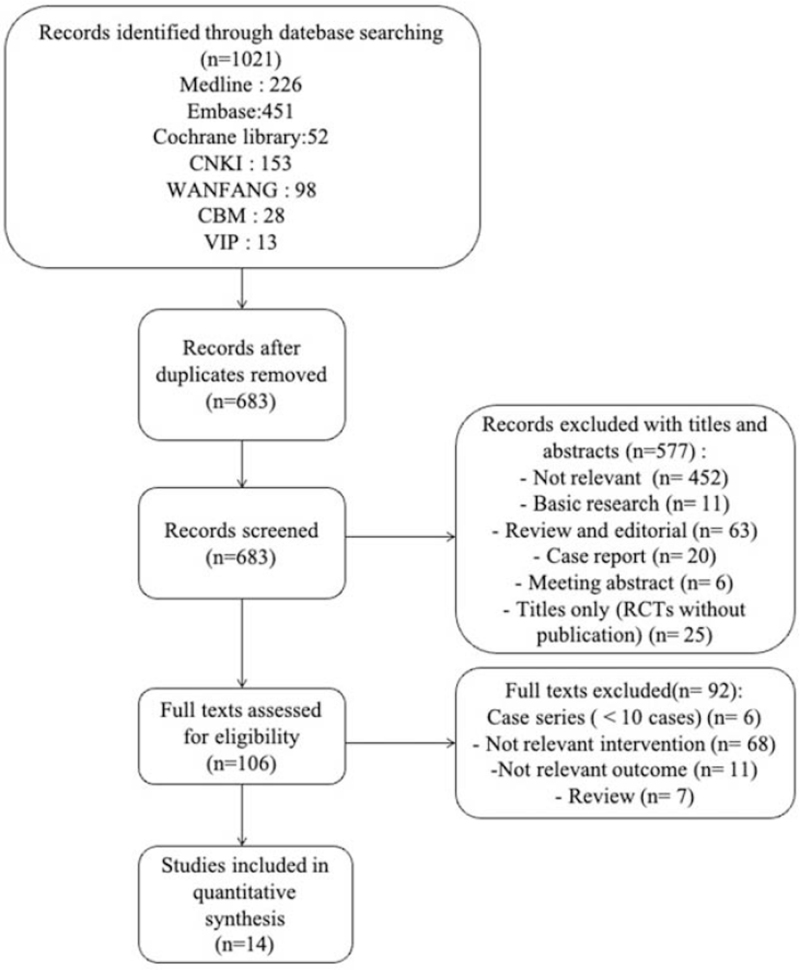
Flow diagram of publication selection.

Our initial search yielded 1021 records and reduced to 683 after removing duplicates. We then excluded the records with only the title and abstract, and 106 were remained. We further excluded 92 studies with less than 10 cases, no research-related intervention measures, and no research-related outcome events. Ultimately, 14 records were used in the final quantitative synthesis (Fig. 1).

### 3.1 Study characteristics

Table 1 summarizes characteristics of included studies.^[17,23–35]^

**Table 1:** Characteristics of studies reporting coronavirus disease 2019.

Author	Ref	Year	Country	Study design	Study period	Sample size	Age (year, mean ± SD/median[IQR])	Sex (male %)	BMI (kg/m^2^, mean ± SD/median [IQR])	Severity of disease
Llitjos et al	^[23]^	2020	France	Retrospective cohort study	March 19–April 11, 2020	26	68 (51.5–74.5)	77	NA	Severe
Hanif et al	^[24]^	2020	USA	Retrospective series	March 15–April 14, 2020	921	62 (median)	62.3	30.4	All
Nadkarni et al	^[17]^	2020	USA	Cohort study	March 1–April 30, 2020	4389	65 (53–77)	56	28 (25–33)	All
Pesavento et al	^[25]^	2020	Italy	Retrospective study	February 26–April 6, 2020	324	NA	55.9	NA	Non-severe
Musoke et al	^[26]^	2020	USA	Single-center retrospective study	March 1–May 31, 2020	355	66.21 ± 14.21	51	29.71 ± 9.11	All
Jimenez Guiu et al	^[27]^	2020	Spain	Single-center prospective cohort study	During April 2020	57	71.3 ± 12.7	50.9	NA	Non-severe
Paolisso et al	^[28]^	2020	USA	Retrospective cohort study	March 1–April 10, 2020	450	67 (55–79)	63	26 (24–30)	All
Lemos et al	^[29]^	2020	Brazil	Randomized open-label phase II study	Not mentioned	20	56.5 ± 13.08	80	33.5 ± 7.80	Severe
Hsu et al	^[30]^	2020	USA	Retrospective review	February 27–April 24, 2020	468	NA	54.9	NA	All
Cho et al	^[31]^	2020	USA	Single-center retrospective cohort study	March 1–May 13, 2020	158	67.4 ± 14.6	53.8	29.5 ± 7.5	All
Benito et al	^[32]^	2020	Spain	Single-center cohort study	March 9–April 15, 2020	76	NA	67.1	NA	All
Zeng et al	^[33]^	2020	China	Single center retrospective cohort study	February 9–March 9, 2020	274	72 (63.5–80.0)	52.9	NA	Moderate to critical
Giacomelli et al	^[34]^	2020	Italy	Prospective cohort study	February 21–April 20, 2020	233	NA	30.9	NA	All
Secco et al	^[35]^	2020	Italy	Single-center retrospective case series	March 13–April 30, 2020	115	69 (55–78)	67.8	NA	All

IQR=interquartile rang, SD= standard deviation.

Of 14 included studies, 13 were retrospective studies, 1 was randomized clinical trial. Most of the studies were from 7 courtiers among which 6 (42.9%) were from USA, 3 (21.4%) were from Italy, followed by Spain (14.3%), China (7.1%), UK (7.1%), Brazil (7.1%), and France (7.1%). The average and median age of study subjects were both over 60 years old, and 13 articles showed that male patients accounted for more than 50%. The sample size ranged from 26 to 4389.

The comparison group in this study was based on their anticoagulation treatments: with and without anticoagulant therapy, and therapeutic and preventive anticoagulant therapy.

### 3.2 Bleeding events

**Figure 2:**
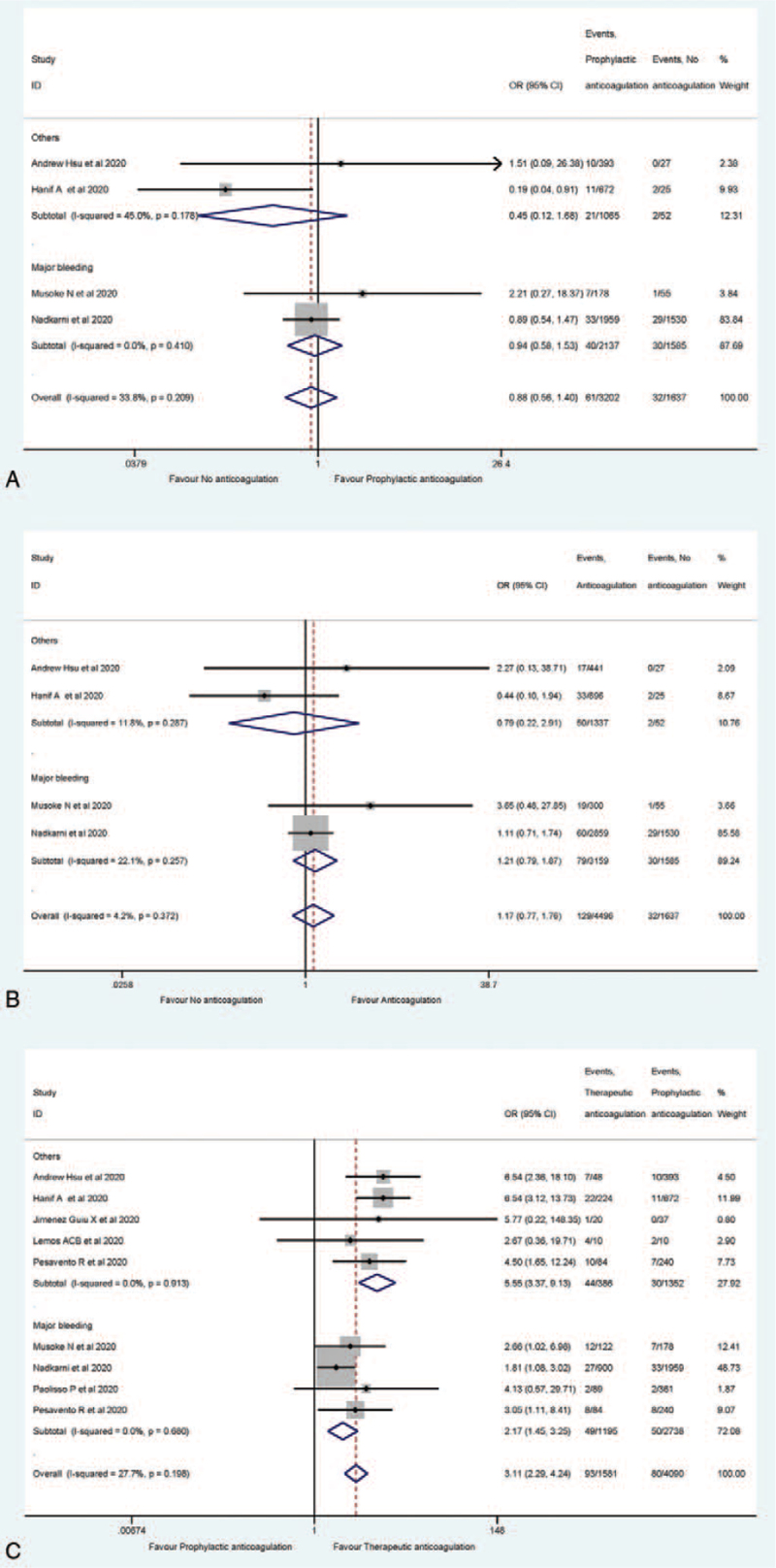
Meta-analysis of the bleeding risk comparison (A for prophylactic anticoagulated vs no anticoagulated; B for anticoagulated vs no anticoagulated; C for prophylactic anticoagulated vs therapeutic anticoagulated).

Table 2 summarizes the outcome indicators. There were 8 studies reporting bleeding event rate for the COVID-19 patients receiving anticoagulation.^[17,24–30]^ In general, there was no increasing trend of bleeding occurrence among those who received anticoagulation. The random efforts model shown that no significant difference between the cohorts with no anticoagulation and prophylactic anticoagulation (Fig. 2A). The same trend was found in the comparison between no anticoagulation and anticoagulation group (Fig. 2B). However, compared with those who received prophylactic anticoagulation, patients with therapeutic anticoagulation had 2.17 times (95% confidence interval [CI]: 1.45–3.25) higher risk ratio of major bleeding rate, and 5.55 times higher risk ratio of other types of bleeding rate. Considering the general bleeding incidence rate, patients with therapeutic anticoagulation had 3.11 times (95% CI: 2.29–4.24) higher bleeding rate than that of patients with prophylactic anticoagulation (Fig. 2C).

**Table 2:** Characteristics of studies reporting coronavirus disease 2019 associated coagulation disorder and clinical outcomes.

					Types of interventions (n)			Number of outcomes (n)		
Author	Ref	Year	Country	Sample size	Therapeutic anticoagulation	Preventive anticoagulation	No anticoagulation	Bleeding events	Thrombosis event	Mortality
Llitjos et al	^[23]^	2020	France	26	18	8	0	NA	18	3
Hanif et al	^[24]^	2020	USA	921	224	672	25	46	NA	543
Nadkarni et al	^[17]^	2020	USA	4389	900	1959	1530	122	NA	1497
Pesavento et al	^[25]^	2020	Italy	324	84	240	0	17	8	NA
Musoke et al	^[26]^	2020	USA	355	128	217	10	27	NA	NA
Jimenez-Guiu et al	^[27]^	2020	Spain	57	12	37	8	1	16	NA
Paolisso et al	^[28]^	2020	USA	450	89	361	0	4	NA	79
Lemos et al	^[29]^	2020	Brazil	20	10	10	0	6	NA	NA
Hsu et al	^[30]^	2020	USA	468	48	393	27	27	43	141
Cho et al	^[31]^	2020	USA	158	14	144	0	NA	88	NA
Benito et al	^[32]^	2020	Spain	76	1	66	9	NA	32	NA
Zeng et al	^[33]^	2020	China	274	Not mentioned	66	Not mentioned	NA	NA	36
Giacomelli et al	^[34]^	2020	Italy	233	233	0	0	NA	NA	48
Secco et al	^[35]^	2020	Italy	115	48	64	3	NA	NA	18

### 3.3 Thrombosis events

**Figure 3:**
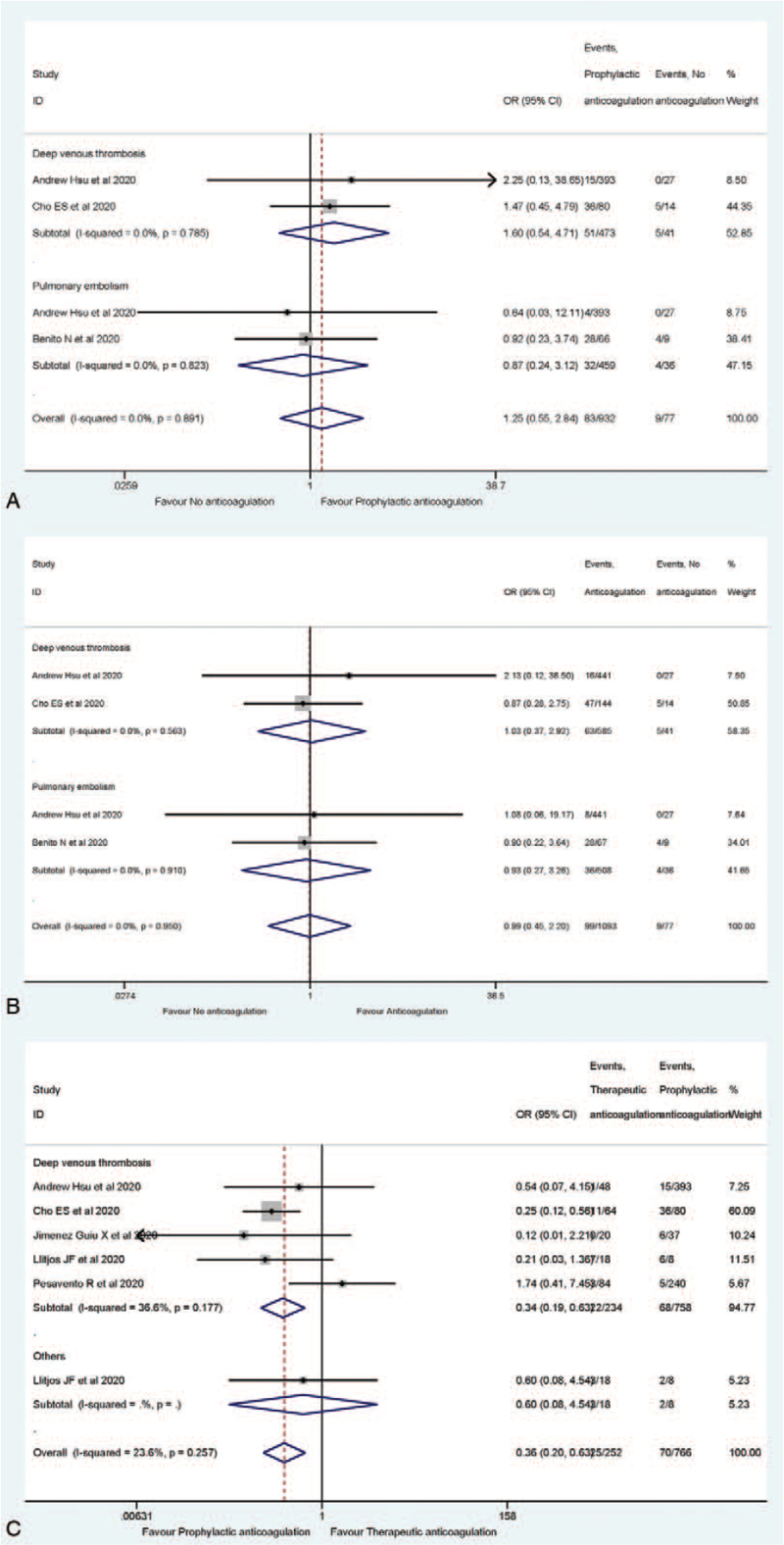
Meta-analysis of the thrombosis risk comparison (A for prophylactic anticoagulated vs no anticoagulated; B for anticoagulated vs no anticoagulated; C for prophylactic anticoagulated vs therapeutic anticoagulated).

According to Table 2, thrombotic events among patients treated with anticoagulation were reported in 6 of the included studies^[23,25,27,30–32]^ and its rate ranged from 2.47% to 69.23%. The percentage of deep venous thrombosis ranged from 6.62% to 55.70%, and that of pulmonary embolism ranged from 2.56% to 42.11%. Specifically, Llitjos et al^[23]^ reported the overall rate of peripheral VTE as high as 69%. Nevertheless, Hsu et al,^[30]^ Cho et al,^[31]^ and Benito et al^[32]^ reported no significant difference in thrombosis events among the 2 comparison cohorts. Overall, the synthesis results indicated that the odds of developing deep venous thrombosis and pulmonary embolism were not statistically significant between the cohorts treated with no anticoagulation and prophylactic anticoagulation and was not statistically significant in the comparison between no anticoagulation and anticoagulation (Fig. 3A and B). Moreover, the odds of deep vein thrombosis in therapeutic anticoagulation was 66% (95% CI: 0.19–0.63) lower than that of prophylactic anticoagulation, as shown in Figure 3C.

### 3.4 Mortality event

**Figure 4:**
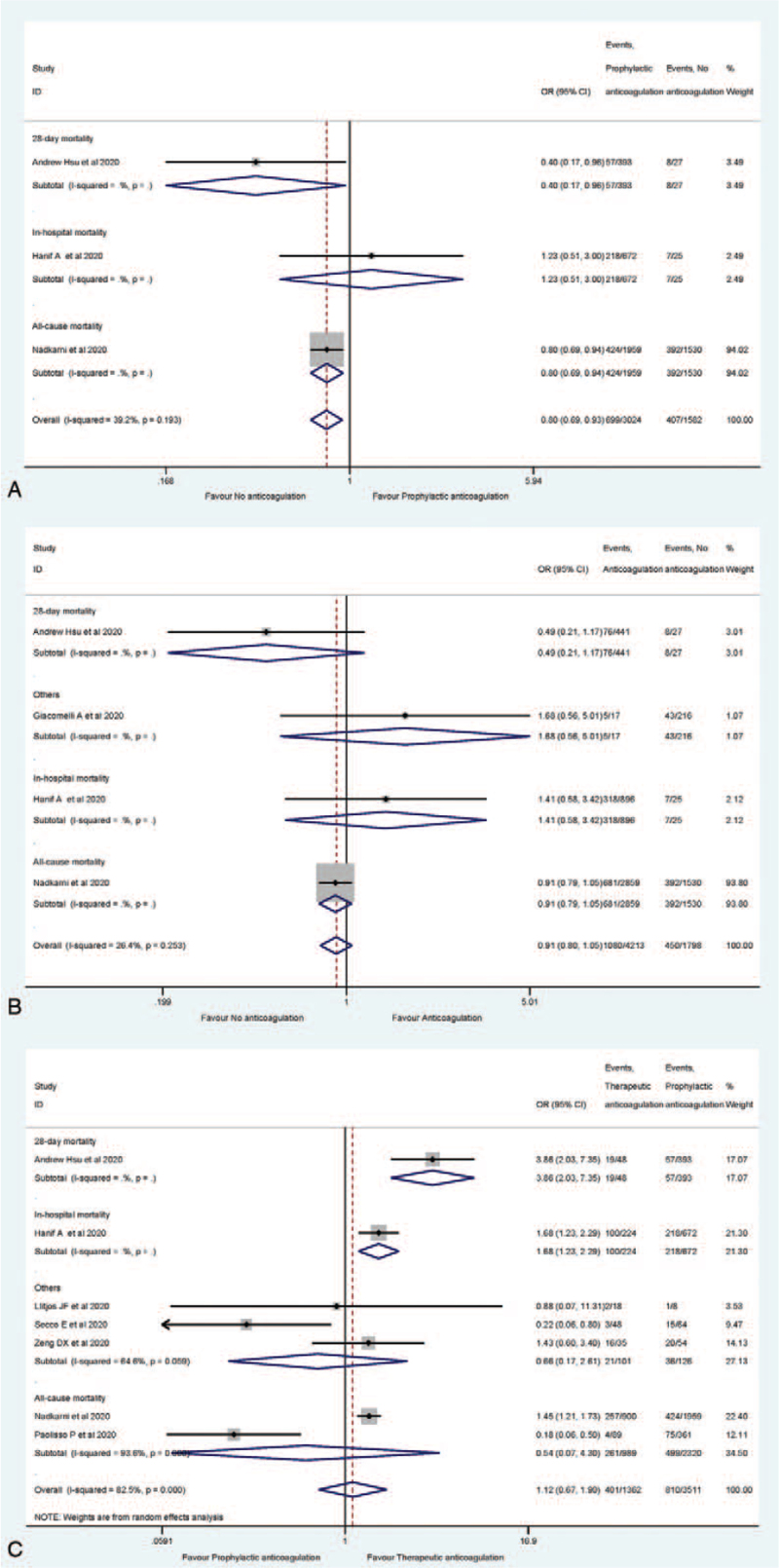
Meta-analysis of the death risk comparison (A for prophylactic anticoagulated vs no anticoagulated; B for anticoagulated vs no anticoagulated; C for prophylactic anticoagulated vs therapeutic anticoagulated).

There were 6 studies that had reported mortality event rate for the COVID-19 patients receiving anticoagulation^[23,25,27,30–32]^ (Table 2). There was basically only 1 paper for each subtype, and the death classification was combined based on the short course of COVID-19. The percentage of all morality event ranged from 11.54% to 58.96%. For all type of mortality regarding 28-day mortality, in-hospital mortality and all-cause mortality, prophylactic anticoagulation group was 20% (95% CI: 0.69–0.93) lower than that of no anticoagulation group, as shown in Figure 4A. In comparison between with and without anticoagulation therapy groups, the trend was consistent with the above (Fig. 4B). Furthermore, no statistical significance was found for general mortality rate between prophylactic anticoagulation and therapeutic anticoagulation (Fig. 4C).

Since substantial heterogeneity was not detected for bleeding, thrombosis or mortality rates, neither among anticoagulated cohort vs the non-anticoagulated cohort, nor preventive anticoagulation cohort vs therapeutic anticoagulation cohort (Figs. 2–4), we found no significant publication bias, as expected, from the funnel plots (Figures S1–S9, Supplemental Digital Content).

## 4 Discussion

Our systematic review and meta-analysis of 14 studies with 7681 COVID-19 patients provided a comprehensive examination of bleeding, thrombosis, and death risk for the presence of anticoagulation treatment, as well as for different types of anticoagulation treatment (prophylactic or therapeutic). The current results indicated that prophylactic or therapeutic anticoagulation was superior to no anticoagulation in reducing mortality. It is worth mentioning that we included 1 short communication from Italy as they also used warfarin or other direct oral anticoagulants, which could be a supplementary route of administration.^[35]^

Persistent evidence has proved that pharmacological thromboprophylaxis can significantly reduce the risk of venous thrombus embolism for general patients.^[36]^ However, the role of prophylactic anticoagulation in COVID-19 patients is still unclear. Some studies revealed the potential benefits of anticoagulant treatment in severe COVID-19 patients with higher venous thrombus embolism risk,^[37–39]^ such as reducing the mortality risk, while other studies indicated that routine chemical prophylaxis is inadequate in preventing venous thrombus embolism in severe COVID-19 patients.^[40]^ In our study, anticoagulation treatment, no matter prophylactic or therapeutic, revealed a mild effectiveness of reducing mortality among COVID-19 patients (prophylactic anticoagulation vs no anticoagulation: OR = 0.80, 95% CI: 0.69–0.93; anticoagulation vs no anticoagulation: OR = 0.91, 95% CI: 0.80–1.05). The results were consistent with the current guidelines advocating treatment and the use of prophylactic and/or therapeutic anticoagulants in patients with COVID-19 appeared to be advocated.^[10,11,41]^ According to the current studies, mechanism of anticoagulant therapy in reduction of thrombosis events among COVID-19 patients might be as follows.^[42,43]^ After infection caused by SARS-CoV-2, the virus would attack angiotensin converting enzyme 2 (ACE2) and decrease its content, making the role of ACE2 from angiotensin II to angiotensin 1 and 7. Therefore, such decrease in ACE2 could lead to an increase in angiotensin II and a decrease in angiotensin 1 and 7, and these changes in angiotensin levels further resulted in an increase in superoxide levels. Recruitment of neutrophils might trigger increasing superoxide production, which contributed to endothelial cell dysfunction through the nitric oxide pathway. Because there were vesicles containing von Willebrand Factor in endothelial cells, when a 500% increase in its activity, these vesicles exulted, and through a number of complex interactions, an increase in von Willebrand Factor could lead to an increase in local thrombus where the inflammation occurred.

Preventive and therapeutic anticoagulant therapy might have opposite clinical effectiveness in reducing bleeding and thrombosis for COVID-19 patients. Venous thromboembolic disease has been reported as one of the major complications occurring in COVID-19 cases^[44]^ without a clear guidance on anticoagulant dose. Jimenez-Guiu et al^[27]^ believed that patients could benefit from intermediate anticoagulation dosages. Bleeding and thrombus were the 2 extremes of coagulation dysfunction.^[45]^ Our results suggested that the risk of bleeding was greater with therapeutic anticoagulation than with prophylactic anticoagulation, while the risk of thrombus was greater with prophylactic anticoagulation than with therapeutic anticoagulation, which might be related to the treatment dose.^[46,47]^ The results supported the need of great attention to drug dosage in anticoagulant therapy, as well as a higher level of evidence-based medicine. Therefore, the therapeutic dose should be carefully considered in clinical practice.

Despite severity of COVID-19 was not stratified in our study, it was adjusted as a confounding factor in a multivariate regression model, higher doses were found to be beneficial for the prevention of death, indicating that the effectiveness value was consistent across different disease degree stratification.^[30]^ Another study showed a much higher frequency of pulmonary embolism in intensive care unit (ICU) patients with COVID-19 (21%) than during the same time interval in 2019 (6%), and it was also higher than the incidence of pulmonary embolism in patients with influenza admitted to the same ICU in 2019 (8%).^[48]^ It seemed to be clear that the incidence of pulmonary embolism in patients admitted to ICU with COVID-19 was much higher than in other critically ill non-COVID-19 patients, including those with acute respiratory distress syndrome and other respiratory infections, despite the fact that these patients were already at an increased risk of pulmonary embolism.^[49]^ In the studies mentioned above,^[40]^ as was the case in the present study, patients developed pulmonary embolism even though most of them were receiving anticoagulant thromboprophylaxis. These findings suggested the great importance of dose administration during anticoagulant therapy in the process of clinical diagnosis and treatment, particularly in higher risk COVID-19 patients.^[6,50,51]^

## 5 Limitations

Our study has several limitations. First, in the outcome event, subgroup analysis usually consisted of only 1 literature, for which we grouped each subtype into a single broad category, including bleeding events, thrombosis events, and death events. Second, literatures included in this study were not stratified according to severity of the disease, so we could not evaluate the clinical benefits for mild and severe patients separately. Third, this study was conducted when the disease outbreak is ongoing, thus many regions affected by COVID-19 have not published clinical datasets, which may skew the results of this analysis. And with all these retrospective datasets, causality was relatively low. Additionally, the small sample size prevented our analysis from subgroups analysis in terms of region and dose. Finally, the meta-analysis was performed by statistical result data, therefore there was no way to analyze case data according to more detailed clinical needs.

## 6 Conclusion

This review provided an anticoagulant therapy strategy for COVID-19 patients. Preventive anticoagulation could effectively reduce the risk of bleeding and pulmonary embolism and therapeutic anticoagulation could reduce the risk of deep venous embolism. Both treatment strategies had significant effectiveness in reducing mortality risk. Our results could shed light on the early anticoagulant therapy among patients with COVID-19.

## Author contributions

Jingyi Ge, Zhipeng Wu and Yingmin Ma conceived the study and designed the protocol. Jingyi Ge and Zhipeng Wu conducted study selection and data extraction. Jingyi Ge, Zhipeng Wu, and Yingmin Ma contributed to statistical analysis and interpretation of data. Jingyi Ge drafted the manuscript with all authors critically revising the manuscript.

**Conceptualization:** Yingmin Ma, Jiawei Jin.

**Investigation:** Jingyi Ge, Yingmin Ma, Jiawei Jin, Xiao Sun.

**Methodology:** Jingyi Ge, Yingmin Ma, Zhipeng Wu, Jiawei Jin, Xiao Sun.

**Supervision:** Yingmin Ma.

**Validation:** Yingmin Ma.

**Writing – original draft:** Jingyi Ge, Zhipeng Wu.

**Writing – review & editing:** Yingmin Ma, Xiao Sun.

## Supplementary Material

Supplemental Digital Content

## Supplementary Material

Supplemental Digital Content

## Supplementary Material

Supplemental Digital Content

## Supplementary Material

Supplemental Digital Content
