# Supplementary material for: Anticoagulation treatment for patients with coronavirus disease 2019 (COVID-19) and its clinical effectiveness in 2020: A meta-analysis study
Source: Medicine (Baltimore). 2021 Nov 24;100(47):e27861. doi: 10.1097/MD.0000000000027861 (PMC8615308; doi:10.1097/MD.0000000000027861)
Supplement: Supplemental Digital Content [file medi-100-e27861-s001.docx]

Supplementary table 2. The Newcastle-Ottawa Scale (NOS) for assessing the quality of studies in meta-analyses

| No. | Study | Design | Selection | | | | Comparability | Outcome | | | Total |
| --- | --- | --- | --- | --- | --- | --- | --- | --- | --- | --- | --- |
| 1 | Pulmonary Thrombosis or Embolism in a Large Cohort of Hospitalized Patients With Covid-19 | Cohort study | * | * | * |  | * | * |  |  | 5 |
| 2 | Utility of D-dimer for diagnosis of deep vein thrombosis in coronavirus disease-19 infection | Cohort study | * | * | * | * | * | * |  |  | 6 |
| 3 | 30-day mortality in patients hospitalized with COVID-19 during the first wave of the Italian epidemic: A prospective cohort study | Cohort study | * | * | * |  | * | * | * |  | 6 |
| 4 | Thrombotic complications and anticoagulation in COVID-19 pneumonia: a New York City hospital experience | Cohort study | * | * | * |  |  | * |  |  | 4 |
| 5 | Intensity of anticoagulation and survival in patients hospitalized with COVID-19 pneumonia | Cohort study | * | * | * |  | ** | * |  |  | 6 |
| 6 | Deep vein thrombosis in nonecritically ill patients with coronavirus disease 2019 pneumonia: deep vein thrombosis in noneintensive care unit patients | Cohort study | * | * | * | * |  | * |  |  | 5 |
| 7 | High incidence of venous thromboembolic events in anticoagulated severe COVID-19 patients | Cohort study | * | * | * | * |  | * | * |  | 6 |
| 8 | Anticoagulation and bleeding risk in patients with COVID-19 | Cohort study | * | * | * | * | ** | * |  |  | 7 |
| 9 | Anticoagulation, Bleeding, Mortality, and Pathology in Hospitalized Patients With COVID-19 | Cohort study | * | * | * | * | ** | * | * |  | 8 |
| 10 | Preliminary Experience With Low Molecular Weight Heparin Strategy in COVID-19 Patients | Cohort study | * | * | * | * | ** | * |  | * | 8 |
| 11 | The hazard of (sub)therapeutic doses of anticoagulants in non-critically ill patients with Covid-19: the Padua province experience | Cohort study | * | * | * | * | ** | * |  |  | 7 |
| 12 | A possible benefit from therapeutic anticoagulation in patients with coronavirus disease 2019: the Dolo hospital experience in Veneto, Italy | Cohort study | * | * | * | * | * | * |  |  | 6 |
| 13 | Association of Padua prediction score with in-hospital prognosis in COVID-19 patients | Cohort study | * | * | * | * | ** | * |  |  | 7 |

Abbreviations: COVID-19= Coronavirus disease 2019. SARS-CoV-2=Severe acute respiratory syndrome coronavirus 2.
