# Supplementary material for: Anticoagulation treatment for patients with coronavirus disease 2019 (COVID-19) and its clinical effectiveness in 2020: A meta-analysis study
Source: Medicine (Baltimore). 2021 Nov 24;100(47):e27861. doi: 10.1097/MD.0000000000027861 (PMC8615308; doi:10.1097/MD.0000000000027861)
Supplement: Supplemental Digital Content [file medi-100-e27861-s002.docx]

Supplementary table 3. Cochrane collaboration tool for assessing the quality of studies in meta-analyses

| Study | Design | Randomization | | Blinding | | | | Patient attrition | | | Total |
| --- | --- | --- | --- | --- | --- | --- | --- | --- | --- | --- | --- |
| Therapeutic versus prophylactic anticoagulation for severe COVID-19: A randomized phase II clinical trial (HESACOVID) | RCT | 1 | 1 | 0 | 0 | 0 | 0 | 1 | 1 | 1 | 5 |

Abbreviations: COVID-19= Coronavirus disease 2019. RCT=Randomized clinical trial.
