# Supplementary material for: Anticoagulation treatment for patients with coronavirus disease 2019 (COVID-19) and its clinical effectiveness in 2020: A meta-analysis study
Source: Medicine (Baltimore). 2021 Nov 24;100(47):e27861. doi: 10.1097/MD.0000000000027861 (PMC8615308; doi:10.1097/MD.0000000000027861)

Supplementary figure 1. Funnel chart-bleeding risk comparison (Prophylactic anticoagulated vs No anticoagulated)


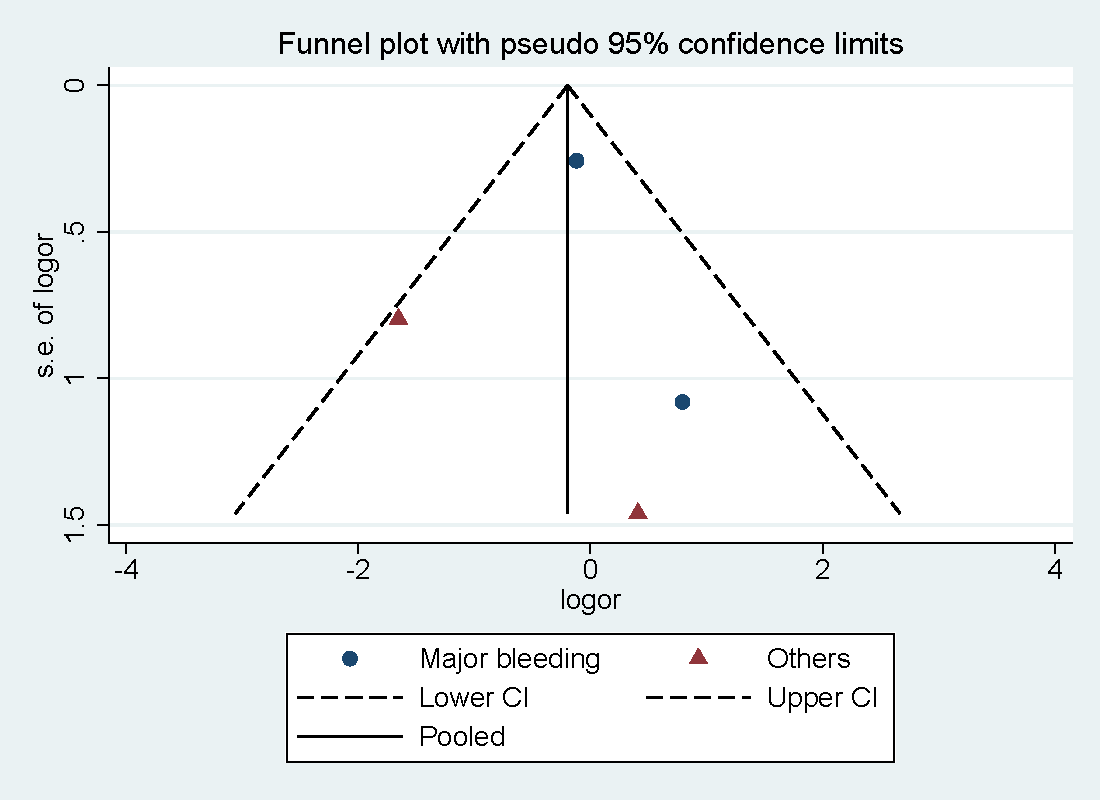


Supplementary figure 2. Funnel chart-bleeding risk comparison (Anticoagulated vs No anticoagulated)


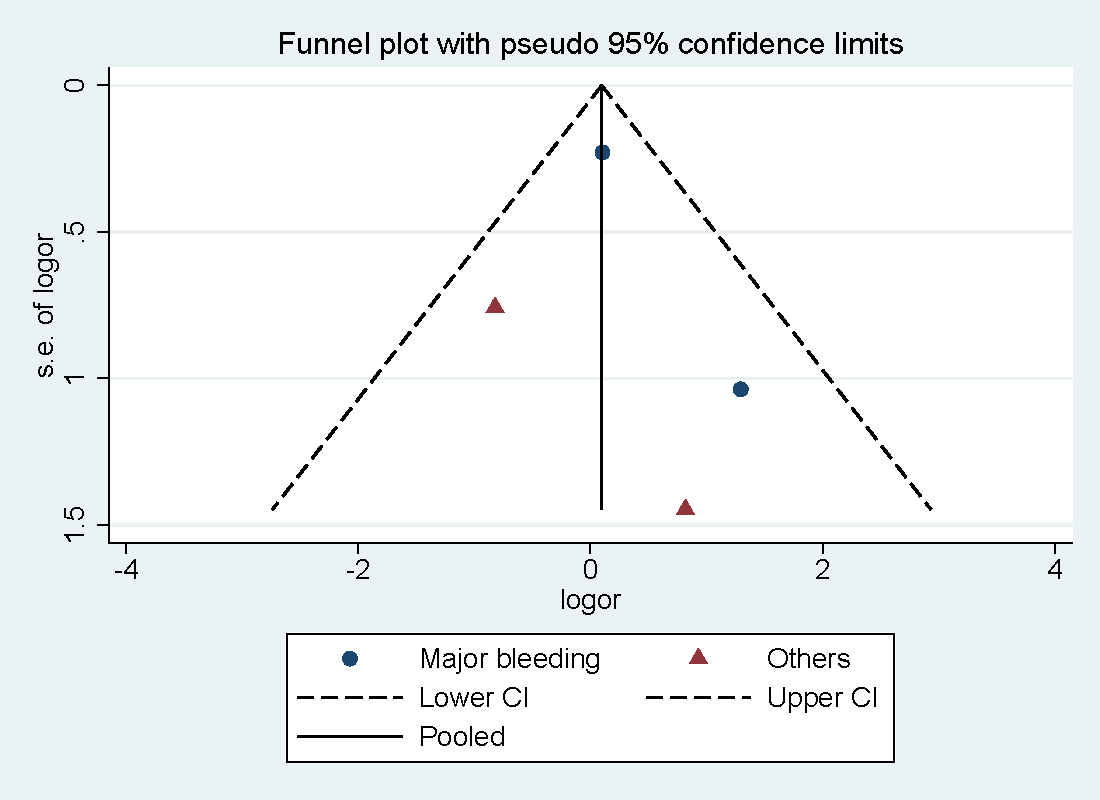


Supplementary figure 3. Funnel chart-bleeding risk comparison (Prophylactic anticoagulated vs Therapeutic anticoagulated)


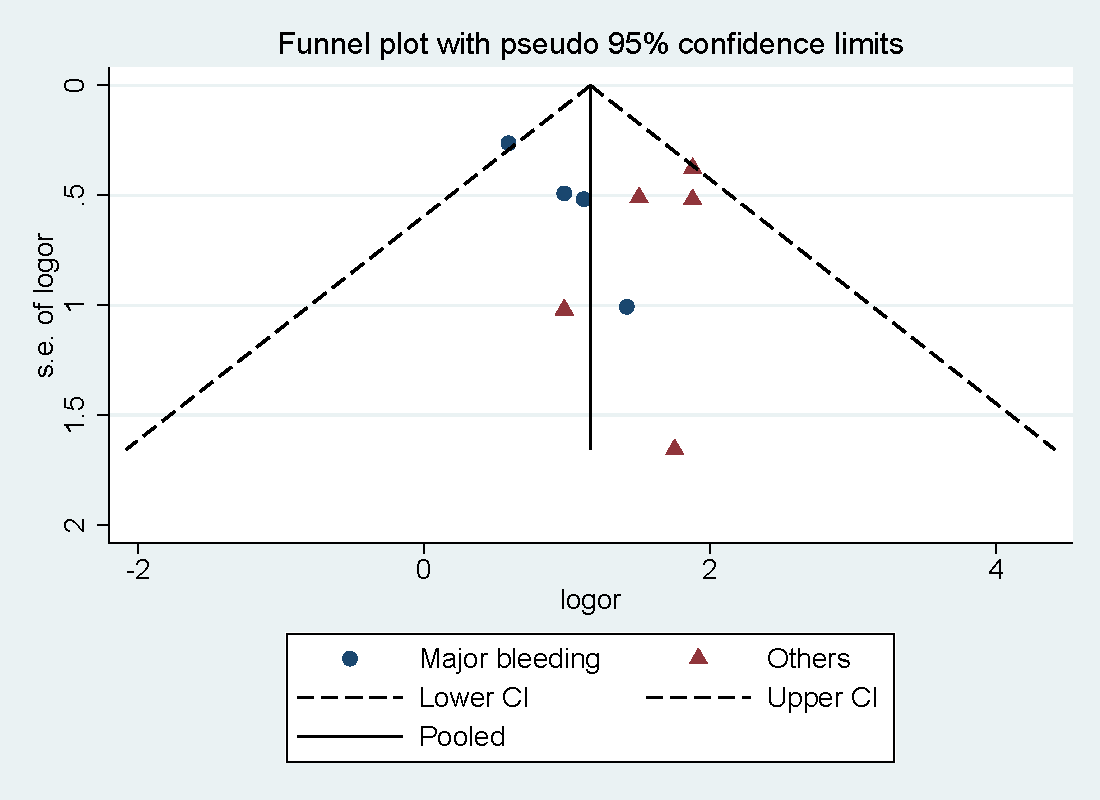


Supplementary figure 4. Funnel chart- thrombosis risk comparison (Prophylactic anticoagulated vs No anticoagulated)


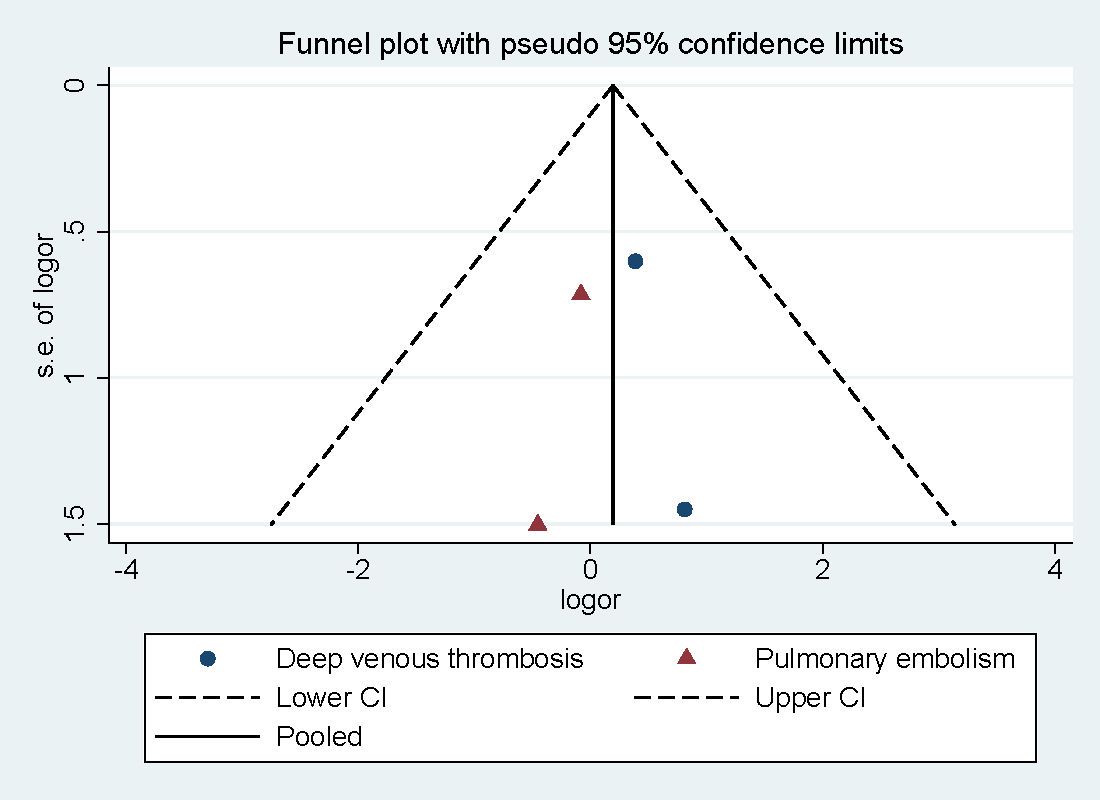


Supplementary figure 5. Funnel chart- thrombosis risk comparison (Anticoagulated vs No anticoagulated)


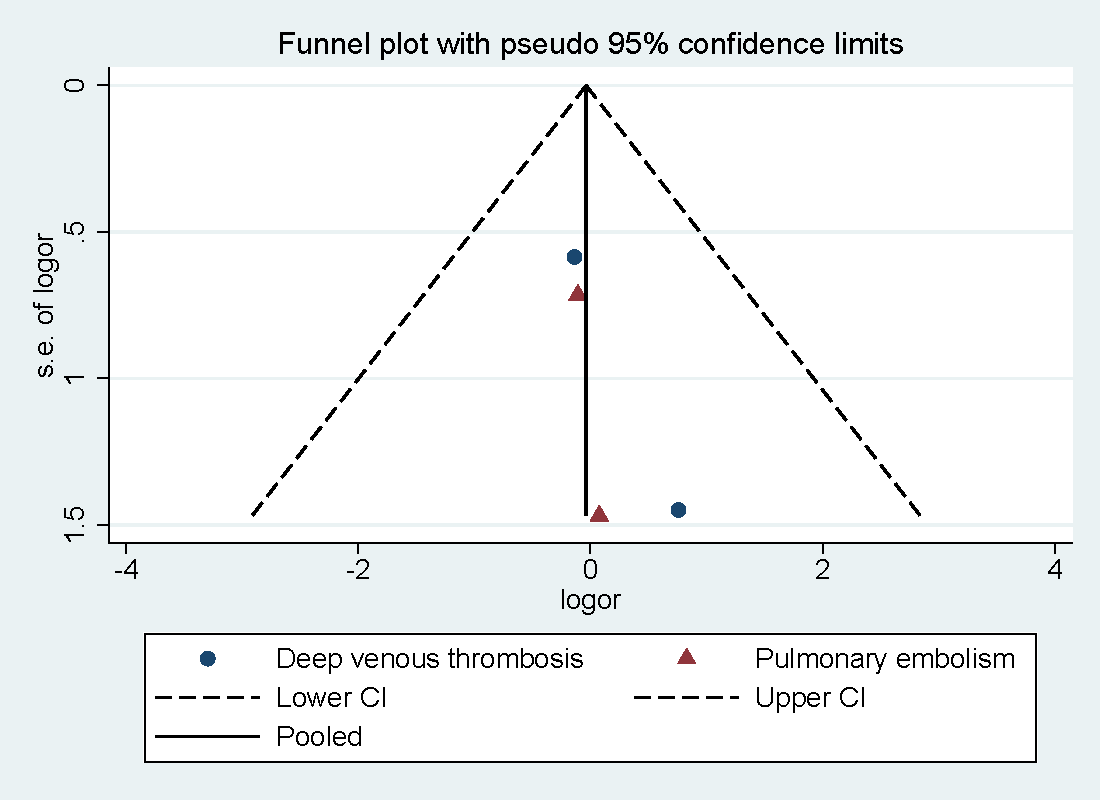


Supplementary figure 6. Funnel chart- thrombosis risk comparison (Prophylactic anticoagulated vs Therapeutic anticoagulated)


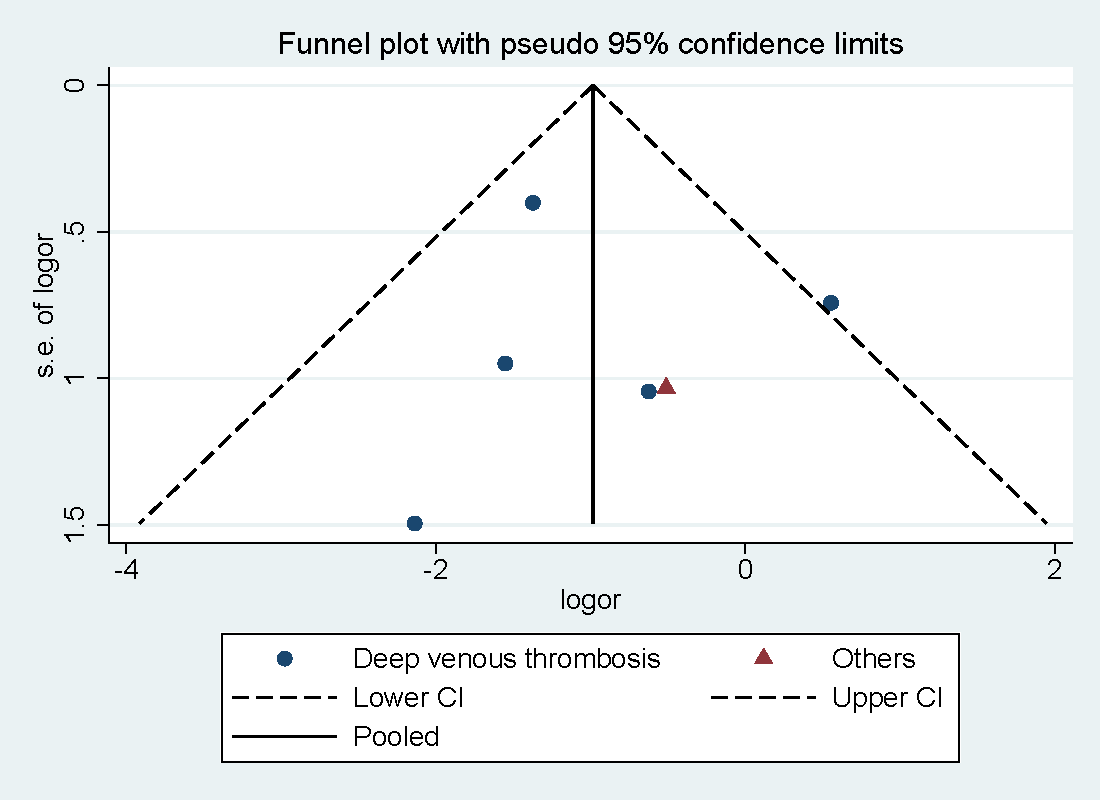


Supplementary figure 7. Funnel chart- death risk comparison (Prophylactic anticoagulated vs No anticoagulated)


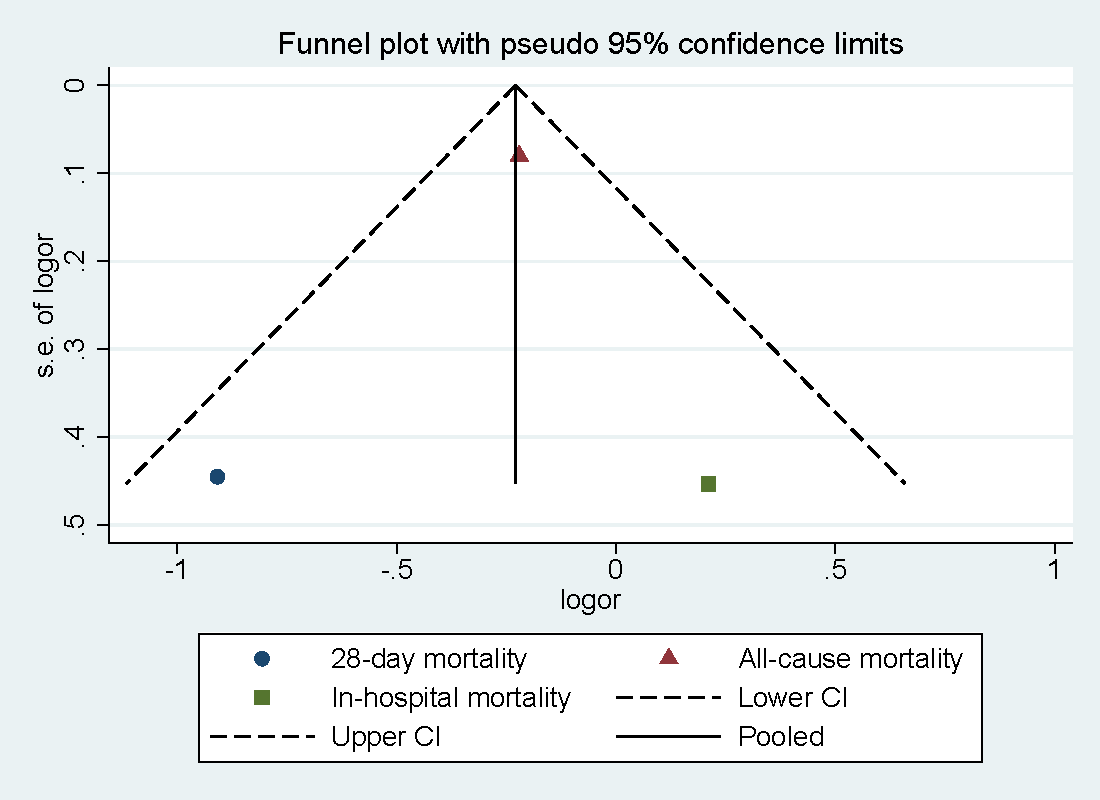


Supplementary figure 8. Funnel chart- death risk comparison (Anticoagulated vs No anticoagulated)


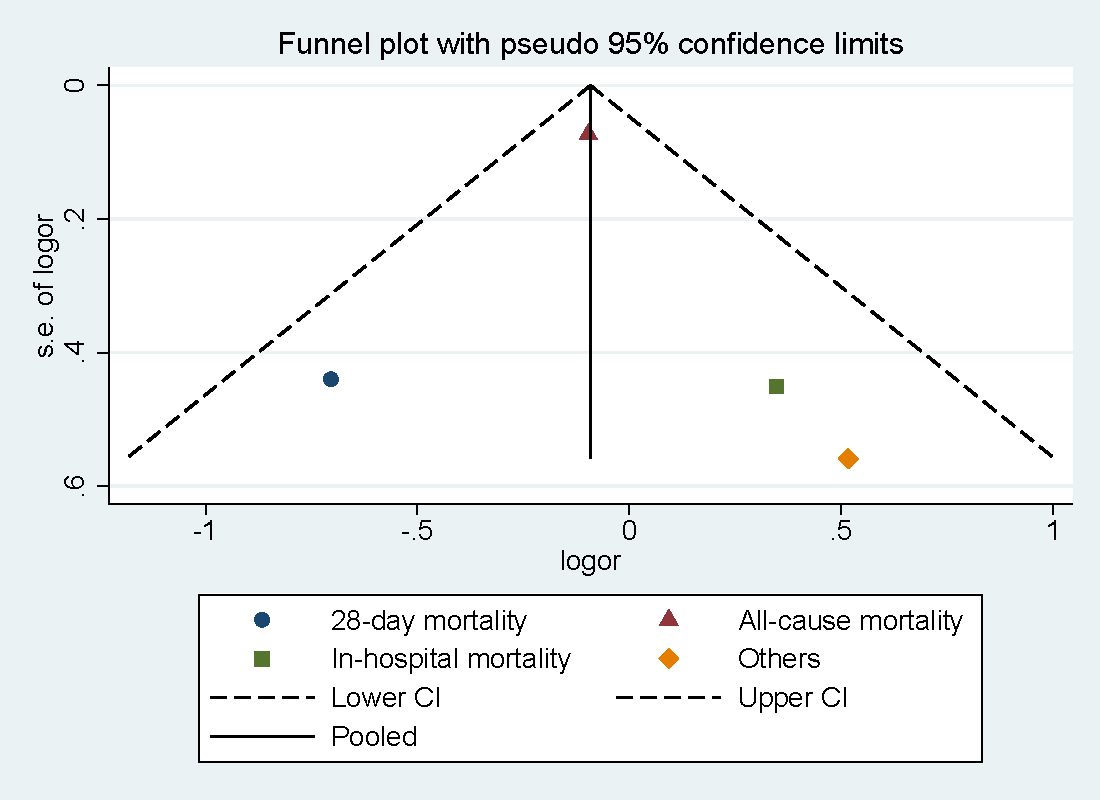


Supplementary figure 9. Funnel chart- death risk comparison (Prophylactic anticoagulated vs Therapeutic anticoagulated)


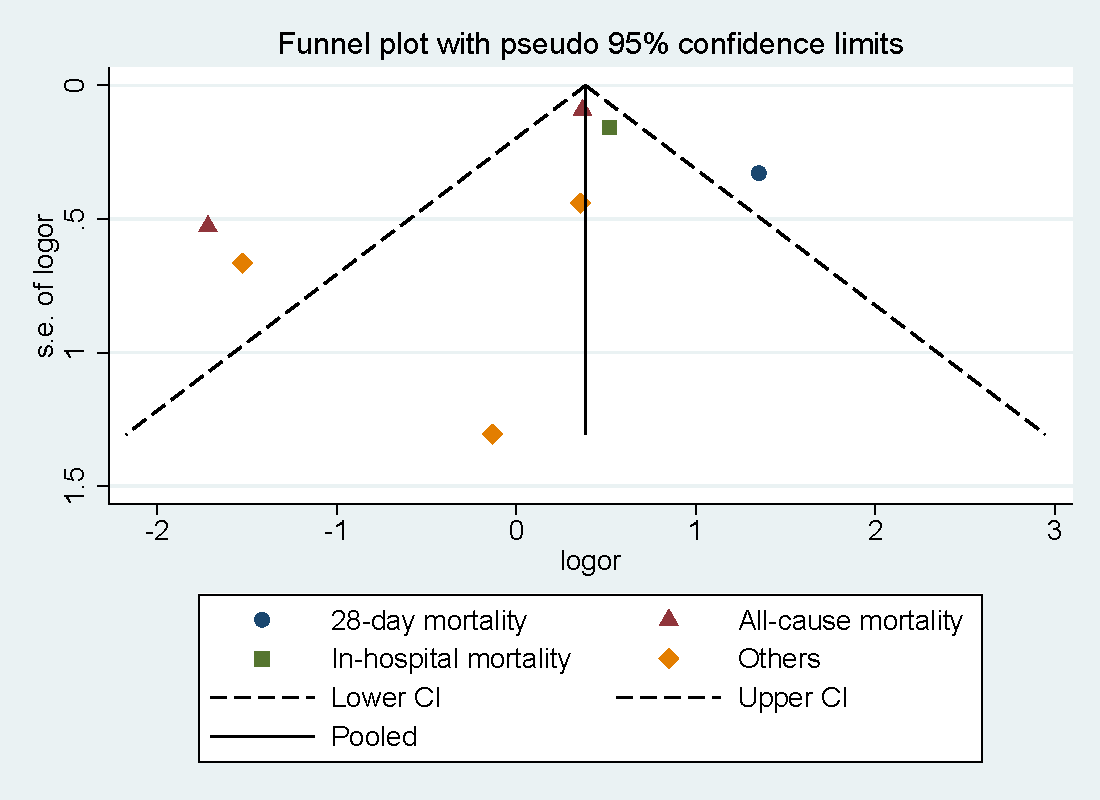

Supplement: Supplemental Digital Content [file medi-100-e27861-s003.docx]
